# Acute glycemic variability and risk of mortality in patients with sepsis: a meta-analysis

**DOI:** 10.1186/s13098-022-00819-8

**Published:** 2022-04-23

**Authors:** Xiaofei Li, Daofu Zhang, Yongxin Chen, Weiwei Ye, Shuang Wu, Lianqing Lou, Yanshuang Zhu

**Affiliations:** 1 Department of Infectious Diseases, Yiwu Central Hospital, No. 519 Nanmen Street, Yiwu, 322000 Zhejiang China; 2 Department of Intensive Care Unit, Liaocheng Daochangfu People’s Hospital, Liaocheng, 252000 China

**Keywords:** Sepsis, Glycemic variability, Mortality, Predictor, Meta-analysis

## Abstract

**Background:**

Acute glycemic variability (GV) has been correlated with the severity of sepsis. The aim of the study was to evaluate the potential association between acute GV and mortality risk in patients with sepsis.

**Methods:**

Cohort studies comparing the risk of death within 3 months between septic patients with higher versus lower acute GV were retrieved by systematic search of Medline, Embase, Web of Science, Wanfang and CNKI databases. We used a random-effect model to pool the data by incorporating the between-study heterogeneity. Sensitivity analyses were performed to evaluate the stability of the findings.

**Results:**

Ten studies including 4296 patients were available for the meta-analysis. Pooled results showed that septic patients with higher acute GV had significantly increased mortality risk compared to those with lower acute GV, as evidenced by results using different parameters including standard deviation of blood glucose (SDBG, risk ratio [RR]: 1.74, 95% confidence interval [CI] 1.36–2.24, p < 0.001; I^2^ = 0%), coefficient of variation of blood glucose (RR: 1.91, 95% CI 1.57–2.31, p < 0.001; I^2^ = 0%), mean amplitude of glycemic excursion (RR: 1.81. 95% CI 1.36–2.40, p < 0.001; I^2^ = 0%), and glycemic lability index (RR: 2.52, 95% CI 1.72–3.68, p < 0.001; I^2^ = 0%). Sensitivity analyses by excluding one study at a time did not significantly affect the results (p all < 0.05).

**Conclusions:**

Higher acute GV may be a predictor of mortality risk in patients with sepsis.

**Supplementary Information:**

The online version contains supplementary material available at 10.1186/s13098-022-00819-8.

## Introduction

Sepsis is a prevalent comorbidity in critically ill patients [1], which is defined as a clinical syndrome that results from the dysregulated inflammatory response to infection that leads to organ dysfunction. Although great efforts have been made regarding the prevention and treatment of sepsis, the incidence of the disease remains high, particularly in high-risk population such as the elderly, the long-term hospitalized patients, and those with innate or acquired immunosuppression [2, 3]. Moreover, the mortality of patients with sepsis is also very high (varying from 30 to 90%), probably because of the complexity of the disease and lack of effective therapeutic strategies [1, 4, 5]. Accordingly, identification of risk factors that are associated with mortality risk in patients with sepsis is important for the improvement of the risk stratification and optimizing the clinical management of these patients [6].

Previous studies showed that glycemic disorders may adversely affect the prognosis in patients with sepsis [7, 8]. For example, both stress hyperglycemia and hypoglycemic events have been related to increased risk of death in patients with sepsis [9, 10]. Interestingly, recent evidence suggests that higher acute glycemic variability (GV), which reflects increased fluctuation in glycemia within or between days, may also be a strong risk factor for mortality in patients with critical illnesses, including sepsis [11–14]. Although no consensus has been reached regarding the standard definition or measuring methods for acute GV, parameters such as standard deviation of blood glucose (SDBG), coefficient of variation of blood glucose (CVBG), mean amplitude of glycemic excursion (MAGE), and glycemic lability index (GLI) have been mostly applied for measuring of acute GV in previous studies [15, 16]. Accumulating evidence has suggested that increased acute GV may be associated with higher mortality risk in patients with sepsis [17–24]. However, the results were not always consistent [25, 26] and a systematic review and meta-analysis according to the different parameters of acute GV have not been performed yet. Therefore, in this study, we performed a meta-analysis to comprehensively evaluate the association between acute GV and mortality risk in adult patients with sepsis.

## Methods

We followed the Preferred Reporting Items for Systematic reviews and Meta-Analyses (PRISMA) statement [27, 28] and Cochrane’s Handbook [29] during the design, performing, and presenting of the meta-analysis.

### Search of electronic databases

We identified studies by a systematic search of Medline, Embase, and Web of Science, China National Knowledge Infrastructure (CNKI) and Wanfang electronic databases using the following terms: (1) "glycemic variability" OR "glyceamic variability" OR "glucose variability" OR "glucose fluctuation" OR "standard deviation of blood glucose" OR "coefficient of variation of blood glucose" OR "glycemic lability index" OR "GLI" OR "mean amplitude of glycemic excursion" OR "MAGE"; and (2) "sepsis" OR "septic" OR "septicemia". Only clinical studies published in English or Chinese were selected. An additional manual check-up for the reference lists of relevant original and review articles were performed as supplement. The last literature search was conducted on October 12, 2021.

### Selection of eligible studies

Inclusion criteria were: (1) cohort studies published as full-length articles; (2) included adult patients (18 years or above) who were admitted for the confirmed diagnosis of sepsis; (3) acute GV was evaluated during hospitalization with one or more parameters including SDBG, CVBG, MAGE, or GLI; (4) incidence of all-cause mortality was reported as outcome of interest and compared between patients with higher versus lower acute GV; and (5) reported relative risk for the incidence of mortality comparing septic patients with higher versus lower acute GV. The definitions of parameters for acute GV were consistent with the criteria applied among the included studies. Specifically, the SDBG calculated as the square-root of the average of the squared differences between individual blood glucose values and the mean [30]. The CVBG was defined as the ratio of the standard deviation (SD) to the mean of blood glucose values during observational periods [30]. The MAGE was calculated by measuring the arithmetic mean of the differences between consecutive peaks and nadirs, provided that the differences are greater than one SD of the mean glucose value [30]. The GLI was calculated as the squared difference between consecutive glucose measures per unit of actual time between the samples [31]. The diagnostic criteria for sepsis were also consistent with the criteria adopted in the original articles. Reviews, preclinical studies, studies that did not include patients with sepsis, studies without the evaluation of acute GV, or studies that did not report mortality in patients with sepsis were excluded.

### Extraction of data and evaluation of study quality

Two of the authors independently conducted electronic database search, extraction of study data, and assessment of study quality according to the inclusion criteria described above. If there were discrepancies, they were resolved by consensus between the authors. The extracted data included the following: (1) name of the first author, year of the publication, study design, country, and clinical settings of the study; (2) population characteristics, including the diagnostic criteria for sepsis, total number, mean age, sex, and proportions of patients with diabetes; (3) parameters used for the evaluating of acute GV, cutoffs for defining of patients with higher versus lower acute GV, and duration of GV measurements; (4) follow-up durations and numbers of patients who dies during follow-up; and (5) variables adjusted when the association between acute GV and mortality outcome was evaluated. The Newcastle–Ottawa Scale [32] was used for study quality assessment, which included three domains such as defining of study groups, between-group comparability, and validation of the outcome. This scale totally scored from 1 to 9 stars, with 9 stars indicating the highest study quality level.

### Statistical methods

Risk ratio (RR) and 95% confidence intervals (CIs) were selected as the general variable for the relationship between acute GV and mortality in patients with sepsis. Data of RRs and standard errors (SEs) were calculated from 95% CIs or p values, and an additional logarithmical transformation was performed to stabilize variance and normalize to the distribution [29]. The Cochrane’s Q test was used to evaluate the heterogeneity, and the I^2^ statistic was also estimated [29]. Heterogeneity was deemed to be significant if I^2^ > 50% [33]. We used a random-effect model for data synthesis because this model has incorporated the potential between-study heterogeneity and could provide a more generalized result [29]. Sensitivity analyses were performed by omitting one individual study at a time to examine the robustness of the finding [29, 34]. The funnel plots were constructed and a visual inspection of the symmetry was conducted to reflect the publication bias [35]. The Egger’s regression asymmetry test was further performed for the evaluation of potential publication bias [29]. We used the RevMan (Version 5.1; Cochrane Collaboration, Oxford, UK) software for the statistical analyses.

## Results

### Results of database search

**Fig. 1:**
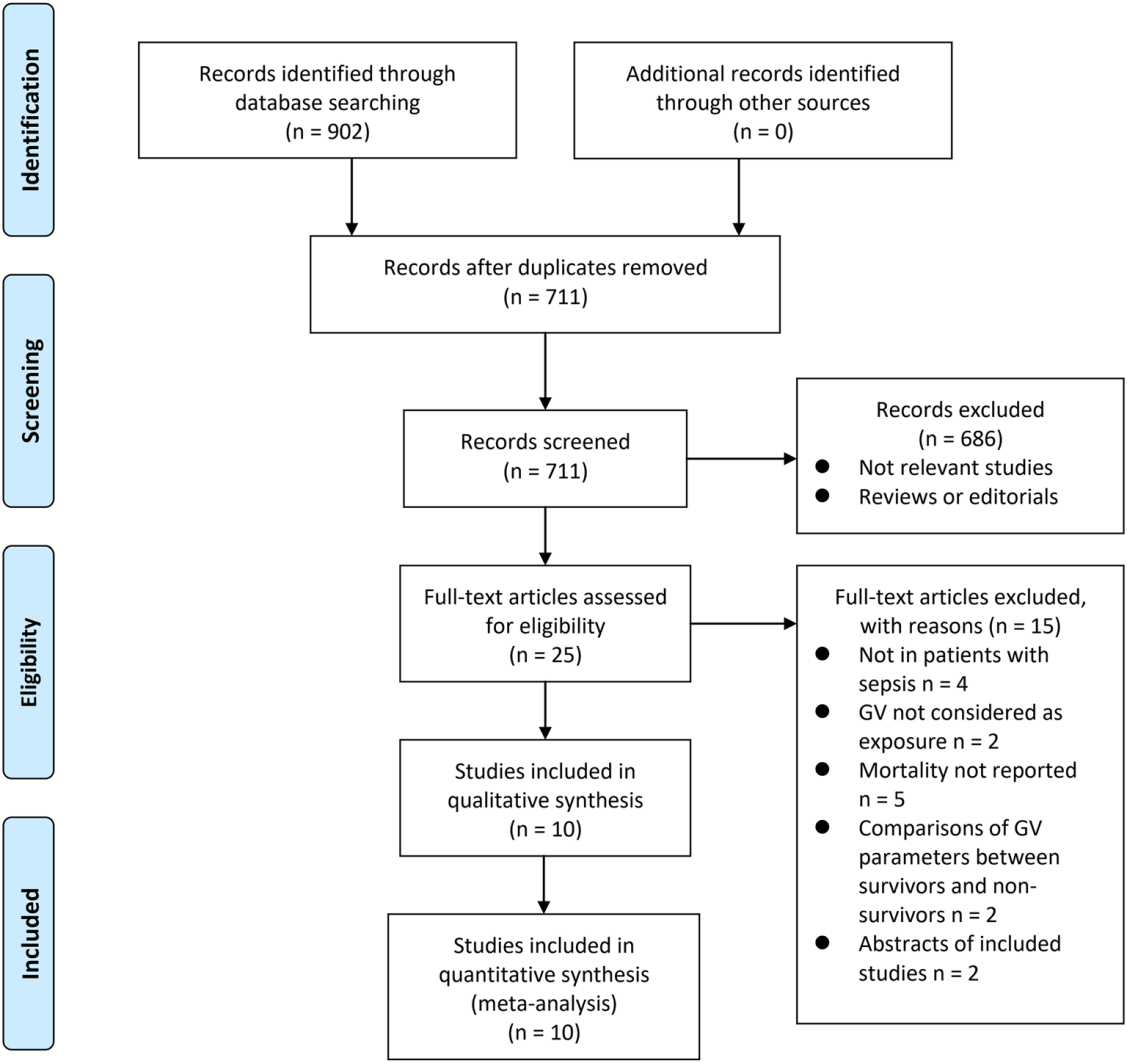
Flowchart of the database search;

The database search process is summarized in Fig. 1. Briefly, 902 articles were found in the initial literature search of the databases; after excluding the duplications, 711 studies remained. An additional 686 were excluded through screening of the titles and abstracts mainly because of the irrelevance to the meta-analysis. The remaining 25 studies underwent a full-text review. Of the 25 studies, 15 were further excluded for the reasons listed in Fig. 1. Finally, ten cohort studies [17–26] were included.

### Characteristics of the included studies

Overall, ten cohort studies including 4296 adult patient with sepsis were available for the meta-analysis, and the characteristics of the studies are shown in Table 1. These studies were published between 2008 and 2021, and performed in China [19–22, 24–26], Japan [23], the United States [17], and Germany [18]. Two of them were prospective cohort studies [18, 23], while the others were retrospective studies [17, 19–22, 24–26]. Diagnosis of sepsis was in accordance with the Sepsis 1.0 criteria in two studies [18, 20], with the Sepsis 2.0 criteria in another two studies [17, 19], and with the Sepsis 3.0 criteria in the remaining six [21–26] studies. The mean age of the included patients varied between 59 and 73 years, and the proportions of males varied from 34 to 88%. The proportions of patients with diabetes were reported in six of the included studies [17, 18, 21, 22, 24, 25]. In five of them, patients with history of DM before admission were recorded [17, 18, 21, 24, 25], while in the other one [22], DM was defined as a diagnosis of DM before admission and those with HbA1c ≥ 6.5% at admission even without a history of DM. Parameters including SDBG [18–20, 24, 26], CVBG [21, 22, 24–26], MAGE [19, 21, 23, 26], and GLI [17, 19, 26] were used for the evaluation of acute GV, and the durations for evaluation of acute GV varied from within the first six hours during ICU stay to during hospitalization. The follow-up durations ranged from within hospitalization to 90 days. A total of 1289 (30.0%) patients died during follow-up. Univariate analyses were applied in two studies for the association between acute GV and mortality in patients with sepsis, while for the other eight studies, multivariate analyses were performed, and variables such as age, sex, the Acute Physiology and Chronic Health Evaluation II (APACHE II) and the Sequential Organ Failure Assessment (SOFA) scores etc. were adjusted. The quality of the included studies were generally good, with NOS varying from six to nine stars (Table 2).

**Table 1:** Characteristics of the included cohort studies

Study	Design	Country	Clinical setting	Diagnostic criteria for sepsis	Sample size	Mean age (years)	Male (%)	DM (%)	GV measurements and cutoff	Duration for GV measurements	Follow-up duration	Death cases	Variables adjusted
Waeschle 2008	PC	Germany	ICU	Sepsis 1.0	191	68	58	29	SDBG (1.1 mmol/l)	During ICU stay	Within hospitalization	46	None
Ali 2008	RC	USA	ICU or general ward	Sepsis 2.0	1246	60	52	32	GLI (medians)	During hospitalization	Within hospitalization	344	Age, sex, hypoglycemia, DM, RF, insulin use, and number of organ failures
Ge 2013	RC	China	ICU	Sepsis 2.0	111	67	70	NR	SDBG, MAGE, and GLI (medians)	During ICU stay	28 days	39	Age and APACHE II score
Wang 2014	RC	China	ICU	Sepsis 1.0	196	59	51	NR	SDBG (median)	During ICU stay	28 days	92	Age, SOFA score at admission, insulin dose, MV, and number of organ failures
Chao 2017	RC	China	ED	Sepsis 3.0	1537	66	54	58	CVBG (30%)	During hospitalization	Within hospitalization	437	Age, sex, DM, comorbidities, BG at admission, and infection sites
Leung 2019	RC	China	ICU or general ward	Sepsis 3.0	317	66	64	46	CVBG (40%)	During hospitalization	Within hospitalization	116	None
Chao 2020	RC	China	ICU	Sepsis 3.0	452	71	77	35	MAGE (3.6 mmol/l) and CVBG (30%)	During ICU stay	30 days	140	Age, sex, HbA1c, severe hypoglycemic episodes, cerebrovascular disease, hemoglobin and creatinine, and APACHE II score
Xu 2021	RC	China	ICU	Sepsis 3.0	73	73	34	NR	MAGE, SDBG, and GLI (medians), and CVBG (30%)	During ICU stay	28 days	18	Age and APACHE II score
Furushima 2021	PC	Japan	ICU	Sepsis 3.0	40	70	88	NR	MAGE (median)	First 48 h in ICU	90 days	11	Age, DM, and APACHE II score
Sun 2021	RC	China	ICU	Sepsis 3.0	133	73	65	24	SDBG (median) and CVBG (20%)	First 6 h in ICU	Within hospitalization	46	Age, SCr at admission, and APACHE II score

DM, diabetes mellitus; GV, glycemic variability; PC, prospective cohort; RC, retrospective cohort; ICU, intensive care unit; ED, emergency department; NR, not reported; SDBG, standard deviation of blood glucose; GLI, glycemic lability index; MAGE, mean amplitude of glycemic excursion; CVBG, coefficient of variation of blood glucose; RF, renal failure; APACHE II, the Acute Physiology and Chronic Health Evaluation II; SOFA, Sequential Organ Failure Assessment; BG, blood glucose; SCr, serum creatinine; HbA1c, glycated hemoglobulin;

**Table 2:** Details of quality evaluation via the Newcastle–Ottawa Scale

Study	Representativeness of the exposed cohort	Selection of the non-exposed cohort	Ascertainment of exposure	Outcome not present at baseline	Control for age	Control for other confounding factors^a^	Assessment of outcome	Enough long follow-up duration	Adequacy of follow-up of cohorts	Total
Waeschle 2008	1	1	1	1	0	0	1	1	1	7
Ali 2008	1	1	1	1	1	0	1	1	1	8
Ge 2013	0	1	1	1	1	1	1	1	1	8
Wang 2014	0	1	1	1	1	1	1	1	1	8
Chao 2017	0	1	1	1	1	0	1	1	1	7
Leung 2019	0	1	1	1	0	0	1	1	1	6
Chao 2020	0	1	1	1	1	1	1	1	1	8
Xu 2021	0	1	1	1	1	1	1	1	1	8
Furushima 2021	1	1	1	1	1	1	1	1	1	9
Sun 2021	0	1	1	1	1	1	1	1	1	8

^a^1 = adjustment for scales that reflect the severity of sepsis, such as the APACHE II (Acute Physiology and Chronic Health Evaluation II) or SOFA (Sequential Organ Failure Assessment) scale; 0 = no adjustment of these scales

### Meta-analysis results

**Fig. 2:**
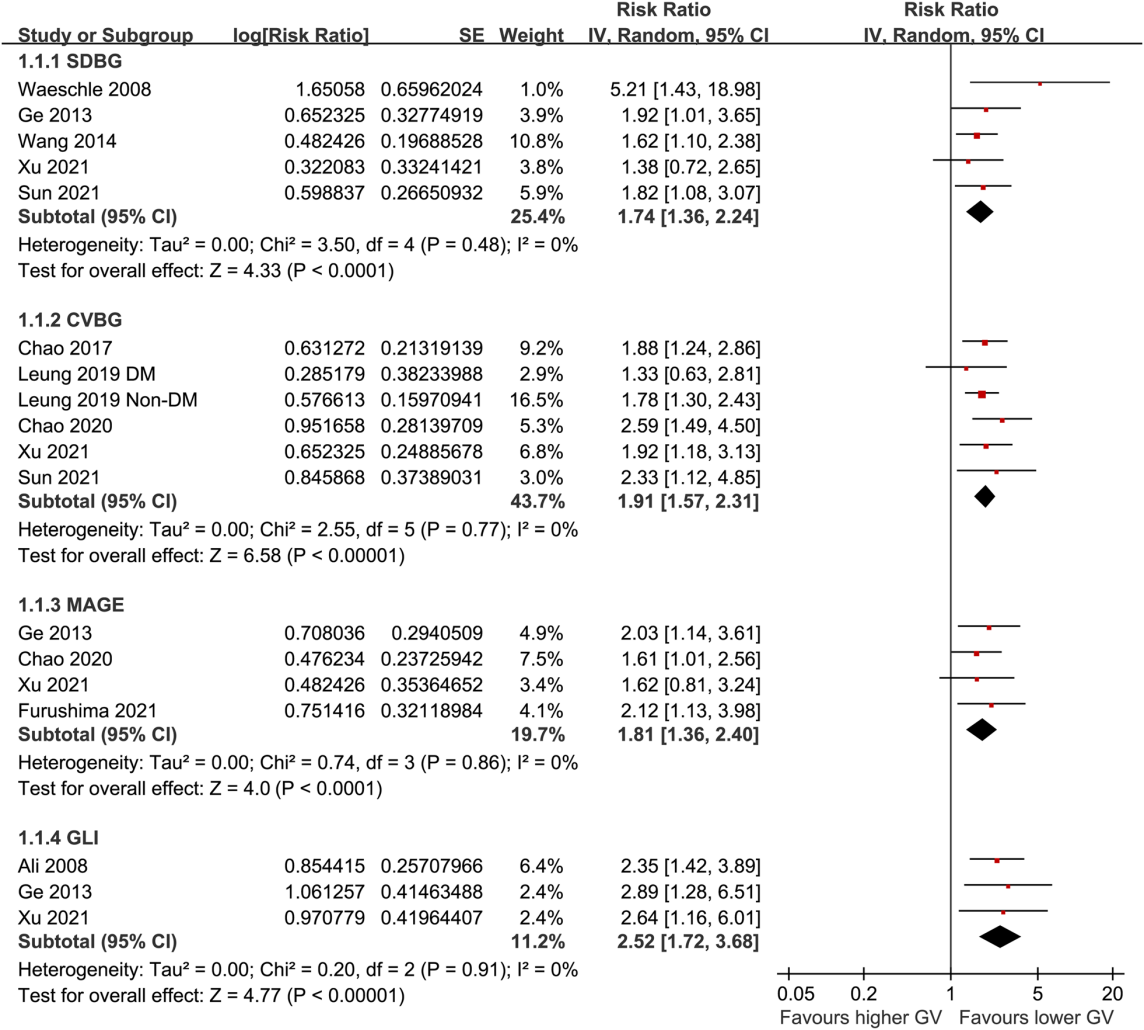
Forest plots for the meta-analyses of the association between acute GV evaluated by SDBG, CVBG, MAGE, and GLI, and the mortality risk in patients with sepsis;

Pooled results of five studies [18–20, 24, 26] showed that higher acute GV evaluated by SDBG was associated with an increased risk of mortality in patients with sepsis (RR: 1.74, 95% CI 1.36–2.24, p < 0.001; Fig. 2) without significant heterogeneity (I^2^ = 0%). Sensitivity analyses by excluding one study at a time showed consistent results (RR: 1.67–1.85, p all < 0.05). Specifically, meta-analysis limited to studies with multivariate analyses showed consistent results (RR: 1.67, 95% CI 1.29–2.16, p < 0.001; I^2^ = 0%).

Pooled results of six datasets from five studies [21, 22, 24–26] showed that higher acute GV evaluated by CVBG was also associated with higher mortality risk in septic patients (RR: 1.91, 95% CI 1.57–2.31, p < 0.001; Fig. 2) without significant heterogeneity (I^2^ = 0%). Sensitivity analyses by omitting one dataset at a time did not significantly change the results (RR: 1.83 to 1.99, p all < 0.05). Further sensitivity analyses limited to studies with multivariate analyses only showed similar results (RR: 2.08, 95% CI 1.61–2.69, p < 0.001; I^2^ = 0%).

Pooled results of four [19, 21, 23, 26] and three [17, 19, 26] studies, all with multivariate analyses, showed that higher acute GV evaluated by MAGE (RR: 1.81. 95% CI 1.36–2.40, p < 0.001; I^2^ = 0%; Fig. 2) and GLI (RR: 2.52, 95% CI 1.72–3.68, p < 0.001; I^2^ = 0%; Fig. 2) were both associated with higher mortality risk in patients with sepsis. Sensitivity by excluding one study at a time showed similar results (for MAGE, RR: 1.73–1.94; for GLI, RR: 2.43–2.76; p all < 0.05).

### Publication bias

**Fig. 3:**
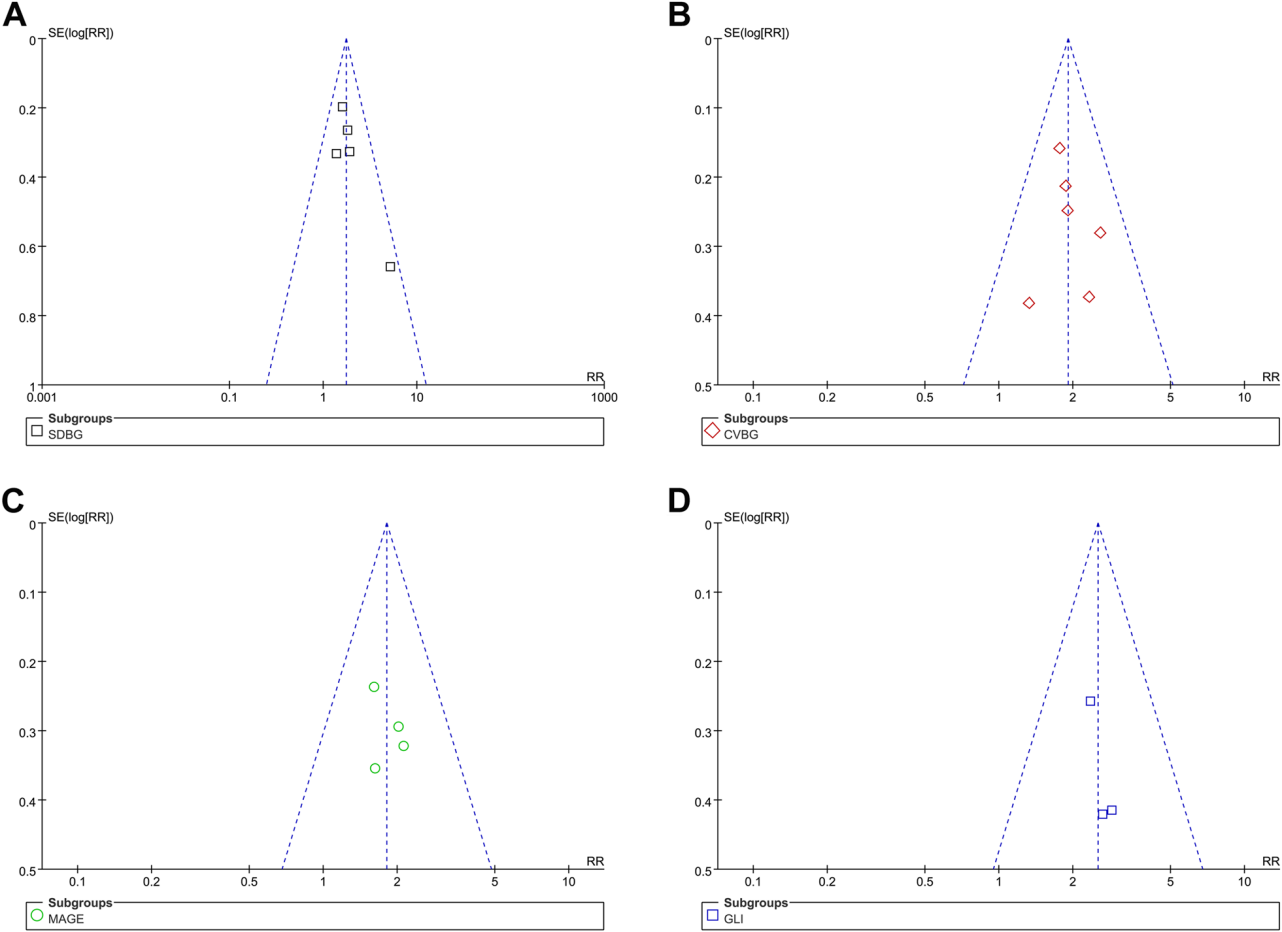
Funnel plots for the publication biases underlying the meta-analyses of the association between acute GV and the mortality risk in patients with sepsis; **A** evaluated by SDBG; **B** evaluated by CVBG; **C** evaluated by MAGE; and **D** evaluated by GLI;

Figure 3 shows the funnel plots regarding the relationship between acute GV evaluated by SDBG, CVBG, MAGE, and GLI with the mortality risk in patients with sepsis. Visual inspection found symmetry of the plots, which suggested low risks of publication biases. The Egger’s regression tests were unable to perform since the limited datasets available for each outcome.

## Discussion

In this meta-analysis, by pooling the results of ten cohort studies, we found that higher acute GV in patients with sepsis is associated with an increased risk of mortality, as evidenced by acute GV measured by parameters including SDBG, CVBG, MAGE, and GLI. Further sensitivity analyses by excluding one dataset at a time did not significant affect the results, and sensitivity analyses limited to studies with multivariate analyses showed consistent results. Taken together, results of this meta-analysis suggested that acute GV may be an independent predictor of mortality in patients with sepsis. Further studies are needed to determine whether incorporating of acute GV into the routine evaluation of patients with sepsis may improve the risk stratification of these patients. Besides, it is also important to explore whether reducing acute GV could improve the prognosis in patients with sepsis.

To the best of our knowledge, this is the first meta-analysis regarding the association between acute GV and mortality risk in patients with sepsis. The strengths of the study include extensive literature search in multiple English and Chinese electronic databases, independent analyses according to the different parameters of acute GV, and application sensitivity analyses to confirm the stability of the findings. Results of the overall meta-analysis consistently showed that higher acute GV, regardless of the different parameters used, is associated with higher risk of mortality in patients with sepsis. Of note, nine of the included studies were performed in Asia [19–26]. Therefore, the results of the meta-analysis were mainly driven by studies from Asia. The only two studies including patients from Europe [18] and the United States [17] both showed a positive association between acute GV and risk of mortality. Accordingly, we believe the results are likely to be generalizable. The possible mechanisms underlying the association between higher acute GV and increased risk of mortality may be multifactorial. Firstly, patients with increased acute GV are more likely to suffer from stress-induced hyperglycemia as well as hypoglycemic events, both of which have been shown to be important predictors of mortality in patients with sepsis [9, 10]. In addition, degree of acute GV may reflect the severity of sepsis. An early study showed patients with severe sepsis and septic shock were more likely to present with higher acute GV [18]. Similarly, another recent study showed that a higher acute GV as measured by MAGE was correlated with the severity of sepsis as evaluated by the SOFA scores [36]. Moreover, it has been well recognized that high glycemic fluctuation is associated with activation of oxidative stress [37] and inflammation [38], two key pathophysiological factors involved in the exacerbation of sepsis and deterioration of subsequent organ function [39]. Besides, findings from recent studies also showed that increased acute glycemic fluctuation is associated with the severity and poor prognosis of other infectious diseases, such as influenza [40] and Coronavirus Disease 2019 (COVID-19) [41, 42].

Although the exact mechanisms and molecular pathways underlying the association between higher acute GV and increased mortality in patients with sepsis remain to be determined, one important question at current stage is that whether the high acute GV is a promising treatment target for sepsis or simply an indicator of disease severity. An early pilot clinical study including 72 Chinese patients with severe acute pancreatitis showed that compared to routine therapy, an additional intensive blood glucose control at 6.1–8.3 mmol/L was associated with reduced glycemic fluctuation, lowered risk of infectious complications, and reduced ICU stay, while the difference between mortality within ICU was not statistically significant [43]. Besides, a recent study showed that minimized glycemic fluctuation was associated with decreased severity and risk of mortality in patients with COVID-19 [44]. Studies are needed to explore whether reducing acute GV could improve the prognosis in patients with sepsis.

Our study also has some limitations. Firstly, the datasets available for evaluating the association between individual parameters of acute GV and the mortality in patients with sepsis were limited. We were unable to determine whether study characteristics, such as study design, demographic factors and comorbidities of patients, and concurrent treatments etc. could affect the association. For example, it has been suggested that obesity [45], diabetic status [46], and some antidiabetic drugs such as metformin [47] may affect the survival outcomes in patients with sepsis. Studies are needed in the future to determine whether difference in these factors may affect the association between acute GV and mortality in patients with sepsis. In addition, difference of the sepsis definition may also affect the outcomes. A post-hoc analysis according to the sepsis definition was shown in Additional file 1: Fig. S1, results of which are of limited value because number of dataset in each subgroup is small. However, the results were consistent for all of the four parameters for acute GV in studies with sepsis defined by current standard (Sepsis 3.0), which may be more important for clinical practice. Moreover, as mentioned previously, no consensus has been reached for the optimal parameters for evaluating acute GV in the critically ill patients. Besides, the cutoff values for defining higher versus lower acute GV for patients with sepsis varied among the included studies, which may also lead to between-study heterogeneity. In addition, eight of the included studies were retrospective studies, while only three were prospective studies. Selection bias related with the retrospective studies may confound the results of the meta-analysis, and large-scale prospective cohort studies are needed to validate these findings. Also, no prospective study involving patients in the general ward was identified, and studies in the future are needed.

Furthermore, although sensitivity analyses limited to studies with multivariate analyses showed similar results, we could not exclude the possible existence of residual factors that may confound the association between acute GV and mortality in patients with sepsis, such as antidiabetic therapies and mean glucose levels. Finally, a causative relationship between cute GV and mortality in patients with sepsis could not be derived based on the findings of the meta-analysis because this is a meta-analysis based on observational studies. Studies should be considered to evaluate whether reducing acute glucose fluctuation could improve the survival in these patients.

## Conclusions

In conclusion, results of this meta-analysis suggested that higher acute GV may be an independent predictor of mortality in patients with sepsis. Studies are warranted to determine the significance of acute GV evaluation for risk stratification of patients with sepsis and to explore whether reducing acute GV could improve the prognosis in these patients.

## Supplementary Information

**Additional file 1: Figure S1.** Influence of difference in the sepsis definitions on the results of the meta-analysis.

## Data Availability

All data generated or analyzed during this study are included in this published article.
