# Supplementary material for: Acute glycemic variability and risk of mortality in patients with sepsis: a meta-analysis
Source: Diabetol Metab Syndr. 2022 Apr 23;14:59. doi: 10.1186/s13098-022-00819-8 (PMC9034073; doi:10.1186/s13098-022-00819-8)

**Additional file 1: Figure S1.** Influence of difference in the sepsis definitions on the results of the meta-analysis


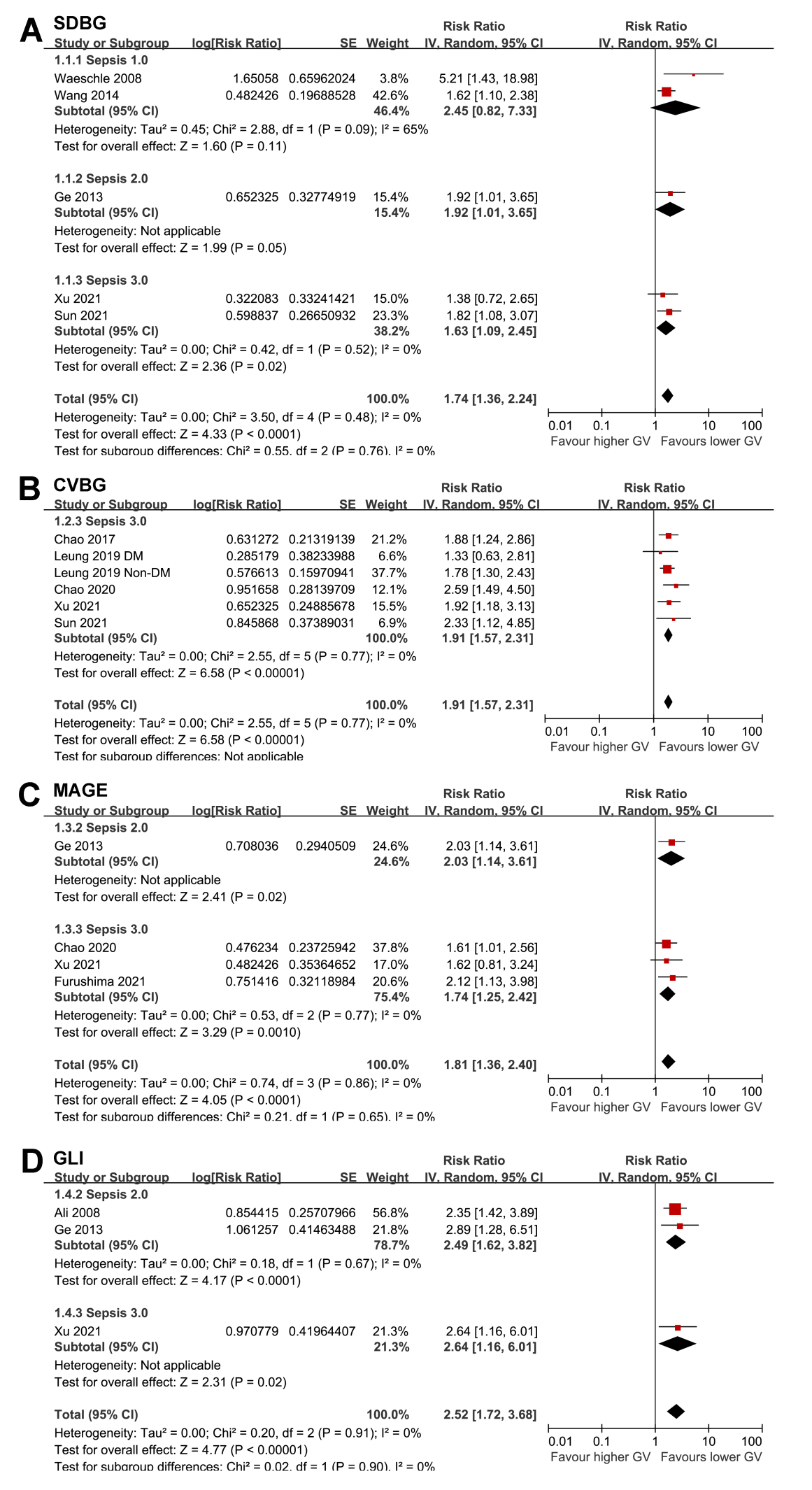

Supplement: Supplementary file 1 — Additional file 1: Figure S1. Influence of difference in the sepsis definitions on the results of the meta-analysis. [file 13098_2022_819_MOESM1_ESM.docx]
